# Quantum Oscillation Signatures of Pressure-induced Topological Phase Transition in BiTeI

**DOI:** 10.1038/srep15973

**Published:** 2015-11-02

**Authors:** Joonbum Park, Kyung-Hwan Jin, Y. J. Jo, E. S. Choi, W. Kang, E. Kampert, J.-S. Rhyee, Seung-Hoon Jhi, Jun Sung Kim

**Affiliations:** 1 Department of Physics, Pohang University of Science and Technology, Pohang, 790-784, Korea; 2 Department of Physics, Kyungpook National University, Daegu, 702-701, Korea; 3 National High Magnetic Field Laboratory, Florida State University, Tallahassee, Florida, 32310, USA; 4 Department of Physics, Ewha Womans University, Seoul, 120-750, Korea; 5 Dresden High Magnetic Field Laboratory, Helmholtz-Zentrum Dresden-Rossendorf, Dresden, D-01314, Germany; 6 Department of Applied Physics, Kyung Hee University, Yongin, 446-701, Korea

## Abstract

We report the pressure-induced topological quantum phase transition of BiTeI single crystals using Shubnikov-de Haas oscillations of bulk Fermi surfaces. The sizes of the inner and the outer FSs of the Rashba-split bands exhibit opposite pressure dependence up to *P* = 3.35 GPa, indicating pressure-tunable Rashba effect. Above a critical pressure *P* ~ 2 GPa, the Shubnikov-de Haas frequency for the inner Fermi surface increases unusually with pressure, and the Shubnikov-de Haas oscillations for the outer Fermi surface shows an abrupt phase shift. In comparison with band structure calculations, we find that these unusual behaviors originate from the Fermi surface shape change due to pressure-induced band inversion. These results clearly demonstrate that the topological quantum phase transition is intimately tied to the shape of bulk Fermi surfaces enclosing the time-reversal invariant momenta with band inversion.

Topological quantum phase transition (TQPT) is a zero temperature transition between distinct topological phases. Unlike conventional phases of matter classified by symmetry breaking^1^, topological phases are defined by topological invariants reflecting a “twist” of bulk electronic wave functions in the presence of an energy gap^2^. When the TQPT occurs by tuning an external parameter like pressure^3^ or chemical composition^4^,^5^, there should be band-gap closing at some points in the Brillouin zone (BZ). At the TQPT, therefore, low energy excitations are described by various types of Dirac dispersions^6^,^7^, offering a fertile ground to test quantum critical phenomena of unconventional relativistic fermions^8^,^9^,^10^,^11^. In this respect, the pressure-induced TQPT is of particular interest. Applying pressure provides a continuous and reversible means to tune electronic structures, which has been widely employed to access closely to the quantum critical point^12^,^13^,^14^. Under pressure, however, the surface-sensitive probes like angle-resolved photoemission spectroscopy cannot be used for detecting the topological surface states as a direct evidence of the TQPT^4^,^5^. Hence experimental identification of the pressure-induced TQPT has remained a challenge so far.

A noncentrosymmetric BiTeI is one of the most interesting candidate systems harboring the pressure-induced TQPT^3^,^15^,^16^,^17^. For the systems with broken inversion symmetry^18^,^19^, low energy excitations at the TQPT are predicted to be a semi-Dirac type, having quadratic dispersion in one direction and linear in the others^6^,^7^. Such intriguing electronic structures have indeed been proposed in a noncentrosymmetric BiTeI at high pressures by recent band structure calculations^3^. Experimental verification, however, remains highly controversial^15^,^16^,^17^. For example, recent optical spectroscopy studies on BiTeI have drawn contradictory conclusions, *i.e.* presence^15^ or absence^16^ of the TQPT at high pressures. Recent studies on pressure-dependent quantum oscillations were also not sufficient to identify the pressure-induced TQPT^20^,^21^, because of the small pressure range, that doesn’t cover the critical pressure^20^ or because of coarse-tuning of pressure that missed the critical pressure^21^. In this paper, we provide experimental evidence of the pressure-induced TQPT in BiTeI by monitoring the Rashba-split bulk FS using magnetic quantum oscillations. From systematic change in Shubnikov de-Haas (SdH) oscillations with pressure up to *P* = 3.35 GPa, we found that the SdH frequency for the inner FS starts to increase unusually with pressure at the critical pressure of *P*_c_ ~ 2 GPa. At the same pressure, the SdH oscillations for the outer FS show an abrupt phase shift. Comparison with band structure calculations reveals that these unusual behaviors arise from the FS shape change due to pressure-induced band inversion across the TQPT. These findings confirm the TQPT in BiTeI at high pressures, and demonstrate that quantum oscillations can provide an effective probe for detecting the pressure-induced TQPT in other candidate systems^22^,^23^,^24^.

## Results

**Figure 1:**
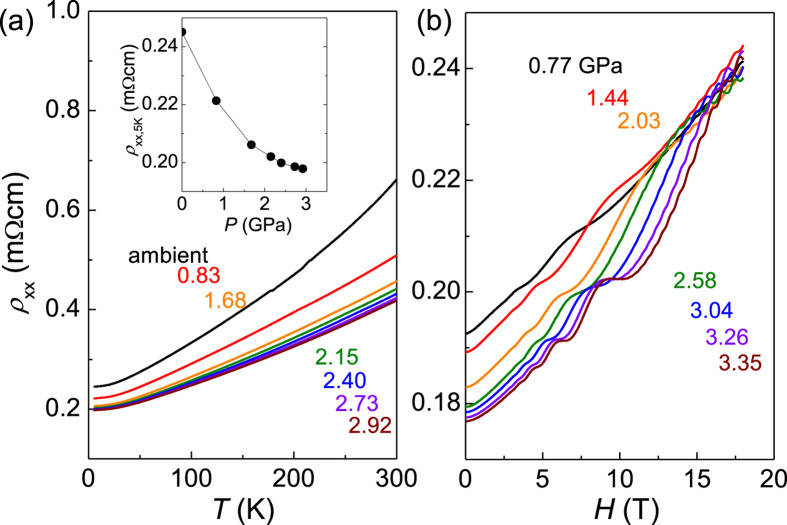
(**a**) Temperature dependence of the in-plane resistivity (*ρ*_*xx*_) at various pressures from ambient pressure to *P* = 2.92 GPa. (**b**) The magnetoresistance for *H//c* taken at different pressures from *P* = 0.77 GPa to *P* = 3.35 GPa.

The temperature dependence of the in-plane resistivity (*ρ*_*xx*_) at zero magnetic field exhibits a systematic decrease in the whole temperature range with increasing pressure. The absolute value of *ρ*_*xx*_ at *T* = 5 K begins to saturate above *P* ~ 2 GPa as shown in the inset of Fig. 1(a). This pressure coincides with the one showing the maximum of the spectral weight of free carriers in a recent optical spectroscopy measurement^15^. The in-plane resistivity *ρ*_*xx*_ under magnetic fields along the *c*-axis also exhibits systematic changes with pressure as shown in Fig. 1(b). Clear SdH oscillations with two distinct frequencies are observed. The well-separated oscillations are due to large difference in size between the inner Fermi surface (IFS) and the outer Fermi surface (OFS) of the Rashba bands^25^,^26^.

**Figure 2:**
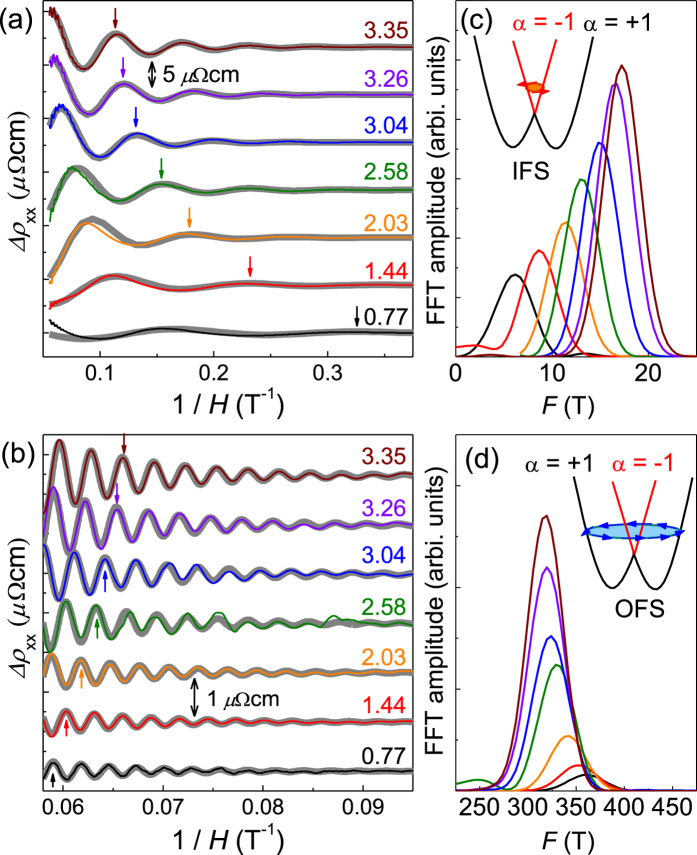
Shubnikov-de Haas oscillations as a function of inverse magnetic field taken at different pressures for (a) the inner Fermi surface and (b) the outer Fermi surface. The background subtracted data were shifted in the *y*-axis for clarity. The fitted curves using the Lifshits-Kosevich formula are presented with grey lines in both (**a**,**b**). The fast fourier transform of the SdH oscillations from the (**c**) inner and (**d**) outer Fermi surfaces. The schematic energy dispersion of Rashba split bands (*α*, the band index) at the *A* point of BZ and the corresponding Fermi surfaces are shown in the inset.

Figure 2(a,b) show the background-subtracted SdH oscillations as a function of inverse magnetic fields for both IFS and OFS. They are well reproduced by fitting to the Lifshits-Kosevich formula with a single frequency as plotted together in Fig. 2. The resistive peak corresponding to the Landau level *n* = 2 for the IFS shifts to higher magnetic fields, while the peak at *n* = 20 for the OFS shifts to lower magnetic fields. Accordingly, the size of the IFS increases, while the OFS shrinks with pressure. This is also confirmed by the fast fourier transform (FFT) as shown in Fig. 2(c,d). From the Onsager relation *F* = (Φ_0_/2π^2^)*S*_F_, where Φ_0_ is the flux quantum and *S*_F_ the Fermi surface size^27^, we found that the size of the IFS (*S*_*F*_^IFS^) increases by 330% up to *P* ~ 3 GPa. For the same pressure range, the size of the OFS (*S*_*F*_^OFS^) decreases only by 12%.

**Figure 3:**
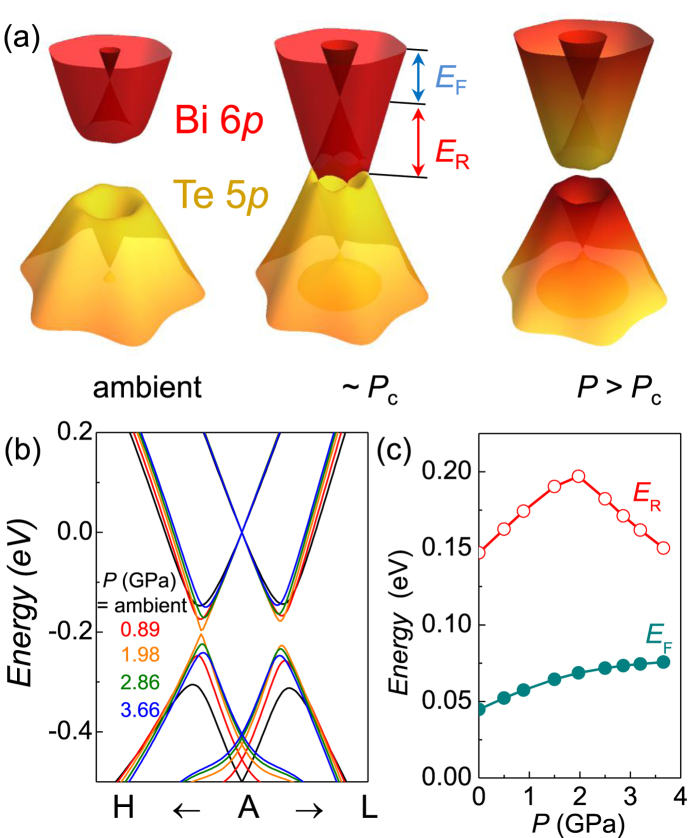
(**a**) Calculated energy dispersion of Rashba bands at various pressures; ambient pressure, 1.98 GPa (*~ P*_c_) and 3.66 GPa from the left to the right. The color code represents the relative band character from the Bi 6*p* and the Te 5*p* states. The Fermi level (*E*_*F*_) and the Rashba parameter (*E*_*R*_) are given relative to the Kramer’s degeneracy point. (**b**) The electronic band dispersion near the *A* point of the BZ at various pressures. Here we fix the *E*_*F*_ at the degeneracy point for a clearer comparison between the band dispersions. (**c**) Pressure dependence of the calculated Rashba energy (*E*_*R*_) and the Fermi level (*E*_*F*_). Here the number of electrons is fixed at ambient pressure where the *E*_*F*_ is set at 45 meV above the degeneracy point.

The opposite pressure dependence of *S*_*F*_^IFS^ and *S*_*F*_^OFS^ is understood from the change of electronic structures upon pressure. As illustrated in Fig. 3(a), the Rashba-split bands from the Bi 6*p* and the Te 5*p* bands form the conduction and valence bands, respectively. With increasing pressure, the overlap of Bi *p*_z_ (Te *p*_z_) states in the neighboring atoms increases. As a result the bandwidth of the conduction (valance) bands is enhanced, leading to the decrease of the FS size at a fixed Fermi level (*E*_*F*_). This is confirmed by band structure calculations. Here we used pressure-dependent volumes and the *c*/*a* ratio taken from experiment^15^. In order to match the inner and the outer FS sizes at the ambient pressure, we set the *E*_*F*_ ~ 45 meV and slightly adjust the *c*/*a* ratio by 0.45%. As shown in Fig. 3(b), the energy dispersion of the OFS changes to pressure, in contrast to the IFS which remains almost the same. The bottom of the Rashba bands is lowered until the band gap is closed on the Γ-*H* symmetry line at *P*_c_ ~ 2 GPa. Above *P*_c_, the band inversion with a gap-opening occurs, consistent with previous calculations^3^. The resulting Rashba energy *E*_*R*_ has a peak at *P*_c_ [Fig. 3(c)], indicating a pressure-tunable Rashba effect.

On the other hands, *E*_*F*_ is increased to conserve the total number of electrons in the system [Fig. 3(c)]. The pressure dependence of *E*_*F*_ was determined by integrating the density of states at a given pressure with the total number of electrons fixed to the value at the ambient pressure. At ambient pressure, *E*_*F*_ of our sample is found to be above the degeneracy point of the Rashba bands^28^. The *E*_*F*_ increases linearly with pressure at a rate of *dE*_*F*_/*dP* ~ 18 meV/GPa up to 2 GPa, and then tends to saturate to ~75 meV [Fig. 3(c)]. For the large-area OFS, the net effect of pressure is dominated by the bandwidth change over the *E*_*F*_ increase. As a result, the OFS shrinks with pressure, consistent with the experiments [Fig. 2(d)]. On the other hand, the small-area IFS is much more sensitive to *E*_*F*_, which expands with pressure as shown in Fig. 2(c).

## Discussion

**Figure 4:**
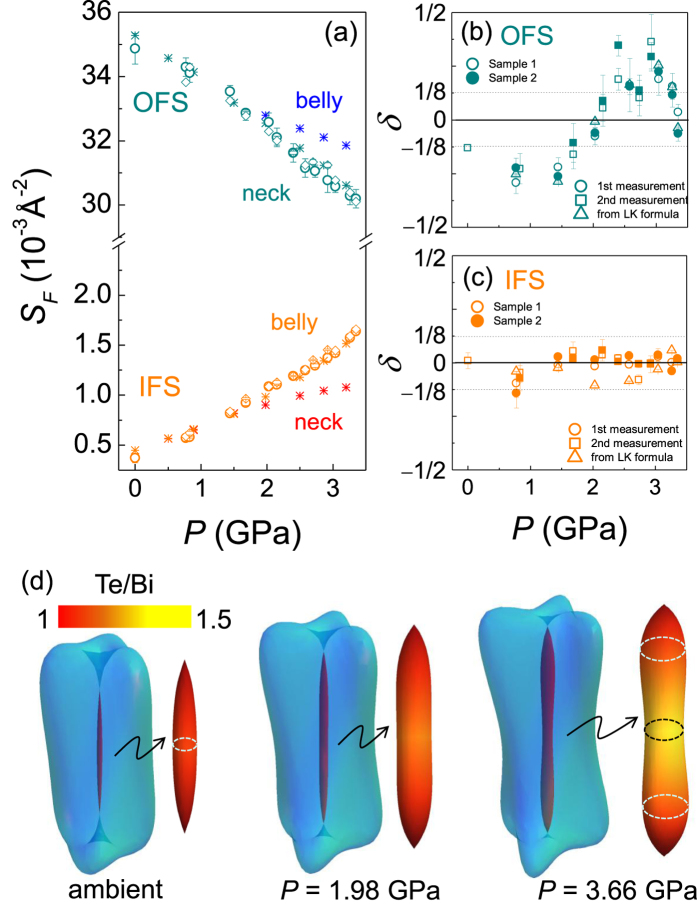
(**a**) Pressure dependence of the Fermi surface size (*S*_*F*_) for the OFS and the IFS taken from the sample 1 (open circles) and 2 (diamonds), as well as from band calculations (asterisks), respectively. Note that the experimental error of pressure in (**a**) is less than the size of the symbol. The error of *S*_*F*_ is the mean absolute error from the FFT analysis. Above *P*_c_ two types of cyclotron orbits, centered at the *A* point (neck) and the other located away from the *k*_*z*_ = *π* plane (belly) in the BZ are plotted. The phase offset *δ* for (**b**) the OFS and (**c**) the IFS as a function of pressure. The open (closed) symbols in (**b**,**c**) represent the data taken from the sample 1 (2). For the sample 1, the phase offset is determined by the Landau fan diagram for the two separate runs (circles and squares), and also by the fitting with the Lifshits-Kosevich formula (triangles) shown in Fig. 2. (**d**) Calculated FS at different pressures: from left, ambient pressure, *P* = 2 GPa (*~ P*_c_), and *P* ~ 3.5 GPa Enlarged IFS is also shown with color denoting the orbital characters. The color bar indicates the ratio (Te/Bi) of the Te 5*p* character to the Bi 6*p* character on the IFS. Circles in dashed lines denote the extremal cyclotron orbits.

Having understood the pressure-dependent FS sizes, we focus on determining the critical pressure *P*_c_ of the TQPT experimentally. Because of the dominant bulk conduction, the topological surface state above *P*_c_ cannot be detected by the transport measurements. Instead, we found that band inversion at the TQPT significantly modifies the bulk FS shape in BiTeI, which can be detected by quantum oscillations. As illustrated in Fig. 3(a), the valence band with dominant Te 5*p* character penetrates into the conduction band of the Bi 6p state at *P*_c_ [Fig. 3(a)]. The changes in relative band character affect the dispersion of Rashba bands and as a result the sizes of FSs. Of particular importance is that the effect of band inversion on the FS sizes is most significant near the time reversal invariant momenta (TRIM), *i.e.* the *A* point in BiTeI. Here the band inversion is maximized in the *A-H-L* plane, but strongly suppressed away from it, as clearly seen by the *k*_*z*_ dependence of the Te 5*p* character on the IFS [Fig. 4(d)]. This induces the FS shape change from the needle- to peanut-type for the IFS. The similar but weaker FS shape change also occurs for the OFS at the TQPT.

Such FS shape changes modify the SdH oscillations^27^. While the needle-shaped FS has a single extremal orbit (belly) centered at *A* point in the BZ, the peanut-shaped FS has two extremal orbits centered at the *A* point (neck) and away from the *A-H-L* plane (belly) [Fig. 4(d)]. If the orbit sizes at the neck and the belly positions are similar, the orbit with a smaller *k*_*z*_ warping gives dominant contribution to SdH oscillations. This is in fact the case for the OFS of BiTeI, where the dominant neck orbit at the *A* point is slightly smaller than the belly orbit by less than 10%, which results a single SdH frequency [Fig. 2(d)]. We found however that the OFS shape change can still be observed by the phase offset *δ* of the SdH oscillations. The phase offset *δ* is determined by the Berry’s phase from the spin texture and also the curvature of the FS in the *k*_*z*_ direction. Having the Berry’s phase constant due to spin chirality in the bulk Rashba bands^29^, *δ* is set by the *k*_*z*_ curvature near the orbit; *δ* = −1/8 (+1/8) for maximum (minimum) extremal cross sections. The phase offset *δ* is estimated either by linear extrapolation of the Landau fan diagram, as shown in Fig. S3 of the Supplementary Material, or by fitting the SdH oscillations with the Lifshits-Kosevich formula, as shown in Fig. 2. We confirmed that the effect of mixing of different transport components (*e*.*g*. *ρ*_xx_ and *ρ*_xy_) is negligible for determining the phase offset *δ* because the quantum oscillations from the longitudinal resistivity (*ρ*_xx_) are in-phase with the longitudinal conductivity (*σ*_xx_ = *ρ*_xx_/(*ρ*_xx_^2^ + *ρ*_xy_^2^)) as shown in Fig. S4 of the Supplemental Material. The contribution of the Zeeman effect is also negligible since any apparent bending is not observed in the Landau level fan diagram for the IFS, and the corresponding Landau levels are far away from the quantum limit (*n* ≥ 18) for the OFS. The estimated *δ* values from different methods are shown for the OFS and the IFS in Fig. 4(b,c), respectively. They are well-matched with each other and also consistent with the results taken from the separate run for the same sample (sample 1) and also for another sample (sample 2). This confirms that the pressure-dependent *δ* indeed reflects the curvature change of the FS. For the OFS, one can clearly notice that the phase offset *δ* changes abruptly near *P*_c_ ~ 2 GPa [Fig. 4(b)]. This indicates the FS curvature of the OFS is changed from the belly-type to the neck-type, which is expected to occur at the critical pressure of the TQPT, as shown in Fig. 4(d), due to band inversion at the *A* points, one of the TRIM point.

Above *P*_c_ ~ 2 GPa, the size of the *S*_*F*_^IFS^ also increases unusually. As shown in Fig. 4(a), *S*_*F*_^IFS^ linearly grows with pressure, but starts to bend upwards at *P*_c_ ~ 2 GPa. The upward behavior of *S*_*F*_^IFS^ cannot be explained by the pressure-dependent *E*_*F*_ that mostly determines the *S*_*F*_^IFS^. As shown in Fig. 3(c), pressure dependence of *E*_*F*_ is linear below *P*_c_ and saturating above *P*_c_. Instead the unusual upturn of *S*_*F*_^IFS^(P) is attributed to the shape change of the IFS near the critical pressure *P*_c_. Above *P*_c_, the belly orbit has a higher SdH frequency than the neck orbit, *e.g.* by 70% at *P* = 3.35 GPa. The calculated *S*_*F*_^IFS^ for the belly position nicely reproduces the upward behavior in experiment. Unlike the OFS, the belly orbit gives dominant contribution to SdH oscillations for the IFS in a whole pressure range as discussed below. This is also consistent with the negligible pressure dependence of the phase offset *δ* [Fig. 4(c)]. Therefore, the unusual increase of the pressure dependent *S*_*F*_^IFS^ also reveals the TQPT at *P*_c_ ~ 2 GPa.

The band inversion at the TQPT also explains the absence of the SdH oscillation from the smaller neck orbit for the IFS above *P*_c_. In BiTeI, there is minute inter-site mixing between Te and I atom due to their similar atomic size and charge. In fact, substitutional point defects of I at Te sites (I_Te_) are known as a source of electron doping for as-grown crystals^30^. This leads to impurity scattering stronger in the FS patch with high Te character. As shown in Fig. 4(d), the cyclotron orbit centered at the *A* point has dominantly Te character above *P*_c_, becoming more susceptible to the I_Te_ defects. This explains the suppressed SdH oscillations in the small orbit near the A point, while larger orbits with mostly Bi character remain intact. These observations suggest that not only the shape of bulk FS but also the relative strength of the quantum oscillations near the band-inversion TRIM point can be a good gauge of detecting the TQPT.

In conclusion, we present experimental evidence of pressure-induced TQPT in noncentrosymmetric BiTeI. From systematic changes of SdH oscillations and band structure calculations, we found that the electronic structure is significantly modified by external pressure and the corresponding Rashba energy is enhanced up to a critical pressure *P*_c_ ~ 2 GPa. The unusual size enhancement of the IFS and the curvature change of the OFS, revealed by SdH oscillations, are understood by the FS shape changes due to the band inversion across the TQPT. These results clearly demonstrate that the band inversion at the TQPT is intimately tied to the FS shape enclosing the TRIM point where the band inversion occurs. Therefore, monitoring bulk FSs using quantum oscillations offers an effective means to identify the TQPT, which can be applied to other candidate systems of the pressure-induced TQPT.

## Methods

### Sample preparation and magnetotransport measurements

The BiTeI single crystals were grown using the vertical Bridgman method^31^. For applying hydrostatic pressure, we used a homemade indenter type pressure cell and a piston-type pressure cell (easyLab Pcell 30, Almax easyLab). The superconducting transition of lead, mounted together with the sample, was used to determine the pressure at low temperatures. Magnetotransport measurements under pressure were done in a four-probe configuration using an 18 T superconducting magnet at the National High Magnetic Field Laboratory, USA. The ambient pressure measurements were done in a pulsed magnet at Hochfeldlabor (HLD), Helmholtz Zentrum Dresden Rossendorf, Germany.

### Band structure calculations

The band structure calculations were carried out in the plane-wave basis within the generalized gradient approximations for exchange-correlation functionals^32^,^33^, using the Vienna *ab-initio* simulation package^34^. A cutoff energy of 400 eV was chosen in the plane-wave basis set to expand the wave-functions and atomic potentials. The k-point integration was done by sampling the Brillouin zone with *k*-point meshes of 10 × 10 × 10 grids. Employing the experimental lattice parameters^15^, the ionic positions were fully optimized until the forces on each ion became less than 0.01 eV/°A.

## Additional Information

**How to cite this article**: Park, J. *et al.* Quantum Oscillation Signatures of Pressure-induced Topological Phase Transition in BiTeI. *Sci. Rep.* **5**, 15973; doi: 10.1038/srep15973 (2015).

## Supplementary Material

Supplementary Materials
