# Supplementary material for: Quantum Oscillation Signatures of Pressure-induced Topological Phase Transition in BiTeI
Source: Sci Rep. 2015 Nov 2;5:15973. doi: 10.1038/srep15973 (PMC4629156; doi:10.1038/srep15973)
Supplement: Supplementary Materials [file srep15973-s1.pdf]

Supplementary Information : Quantum Oscillation Signatures of Pressure-induced Topological Phase Transition in BiTeI

Joonbum Park,[1] Kyung-Hwan Jin,[1] Y. J. Jo,[2] E. S. Choi,[3]
W. Kang,[4] J.-S. Rhyee,[5] Seung-Hoon Jhi,[1] and Jun Sung Kim[1]

[1]*Department of Physics, Pohang University of Science and Technology, Pohang, 790-784, Korea*

[2]*Department of Physics, Kyungpook National University, Daegu, 702-701, Korea*

[3]*National High Magnetic Field Laboratory, Florida State University, Tallahassee, Florida, 32310, USA*

[4]*Department of Physics, Ewha Womans University, Seoul, 120-750, Korea*

[5]*Department of Applied Physics, Kyung Hee University, Yongin, 446-701, Korea*

(Dated: October 29, 2015)

SUPPLEMENTARY 1 : TEMPERATURE DEPENDENCE OF SHUBNIKOV-DE HAAS OSCILLATIONS

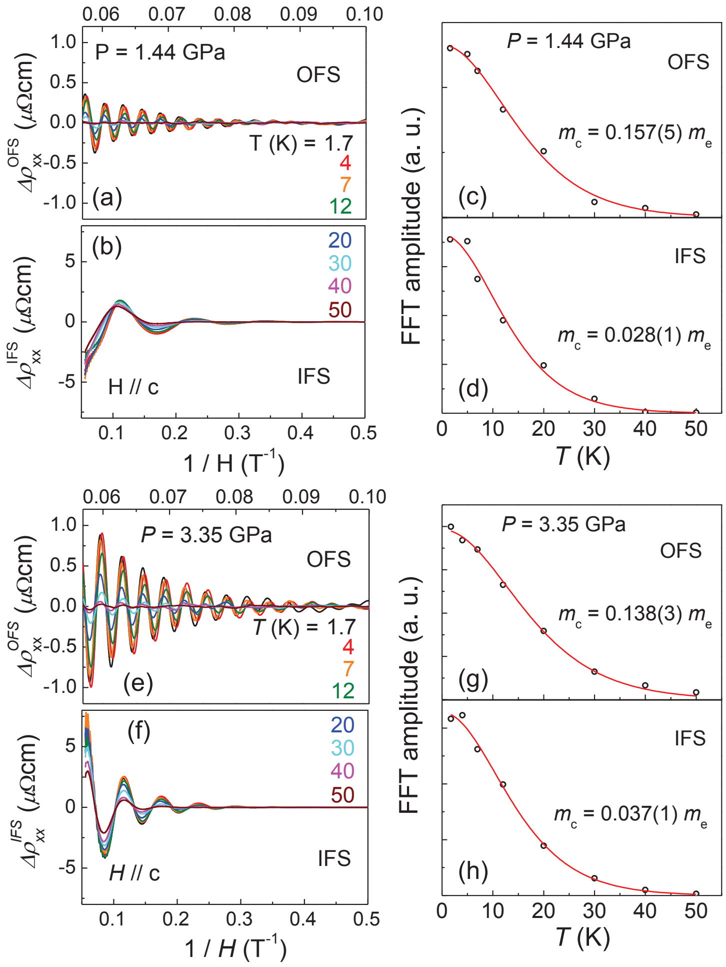

FIG. 1: (color online) Temperature dependence of the background subtracted Shubnikov-de Haas oscillations as a function of the inverse magnetic field (a), (e) for the outer Fermi surface and (b), (f) for the inner Fermi surface at $P = 1.44$ GPa and $P = 3.35$ GPa, respectively. The temperature dependence of the SdH amplitude (c), (g) for the outer Fermi surface and (d), (h) for the inner Fermi surface. The physical parameters of the Fermi surfaces such as the size and the cyclotron mass were extracted using the Lifshitz-Kosevich formula [28].

SUPPLEMENTARY 2 : PRESSURE DEPENDENCE OF THE CYCLOTRON MASS

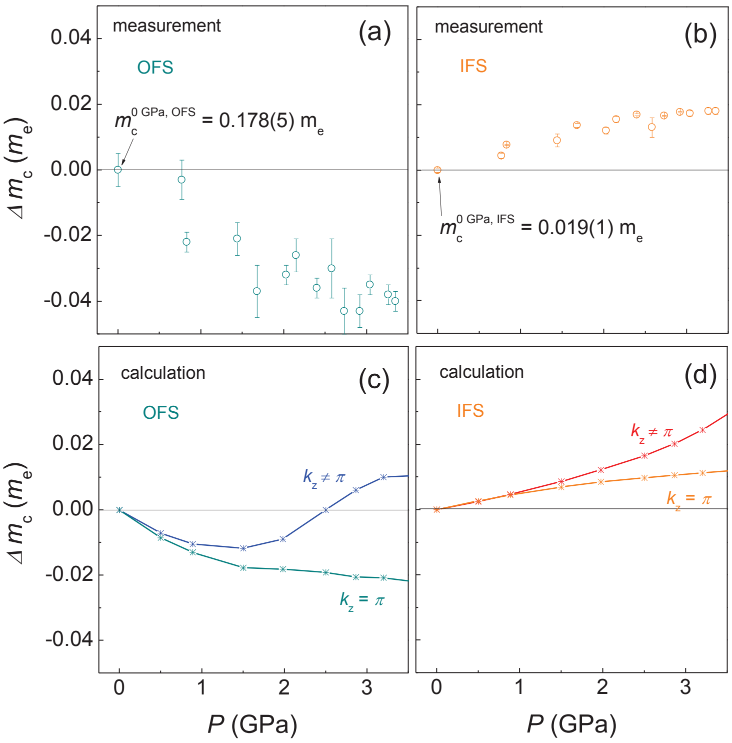

FIG. 2: (color online) Pressure dependence of the cyclotron mass ($\Delta m_c = m_c^{\mathrm{P}}$ - m_c^0) from (a), (b) and measurements and from (c), (d) calculations. The orange circles represent the cyclotron mass from the inner Fermi surface, and the green circles represent the outer Fermi surface. The cyclotron mass of the OFS decreases from $m_c^{\mathrm{ambient}} = 0.178(5)\ m_e$ as the pressure is applied, which is consistent with the calculation results given from the belly of the Fermi surface (blue line, $k_z = \pi$). For the IFS, the cyclotron mass increase from $m_c^{\mathrm{ambient}} = 0.019(1)\ m_e$ with pressure. The m_c of the IFS from the measurement lies between the red and the orange line in (d).

SUPPLEMENTARY 3 : PRESSURE DEPENDENCE OF THE LANDAU LEVEL FAN DIAGRAM

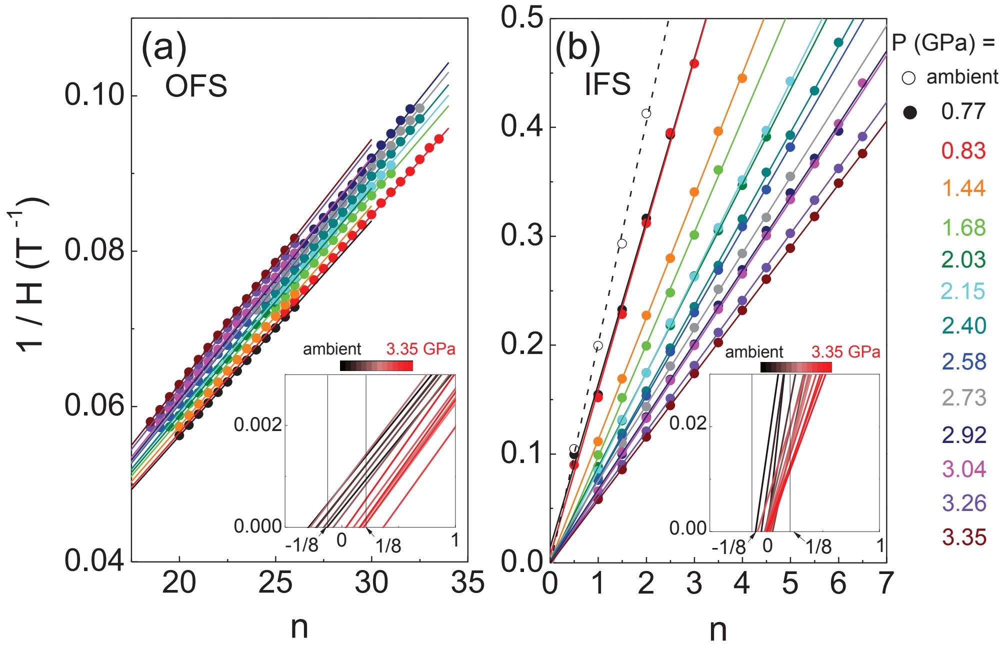

FIG. 3: (color online) Pressure dependence of the Landau level fan diagram for (a) the OFS and (b) the IFS. The solid lines represent the least square linear fits for the Landau level fan diagram at the corresponding pressures. The magnified view of the intercepts of the linear fit to the n-axis, which represent the phase offset, are shown in each insets. The color scale for the insets has been changed in order to signify the effect of pressure on the phase offset. In the OFS, the phase offset shows a noticeable transition from -1/8 to 1/8 as the pressure is increased above the critical pressure. On the other hand, the phase offset for the IFS stays in between -1/8 and 1/8. This is a direct proof that the Shubnikov-de Haas oscillations in the OFS which initially develops from the belly of the Fermi surface at ambient pressure, evolves to develop from the neck of the Fermi surface above the critical pressure. The Shubnikov-de Haas oscillations in the IFS develops from the belly in the whole pressure ranges. These observations are consistent with the orbital character distribution from the band structure calculations.

SUPPLEMENTARY 4 : COMPARISON BETWEEN $\Delta\sigma_{xx}$ AND. $\Delta\rho_{xx}$

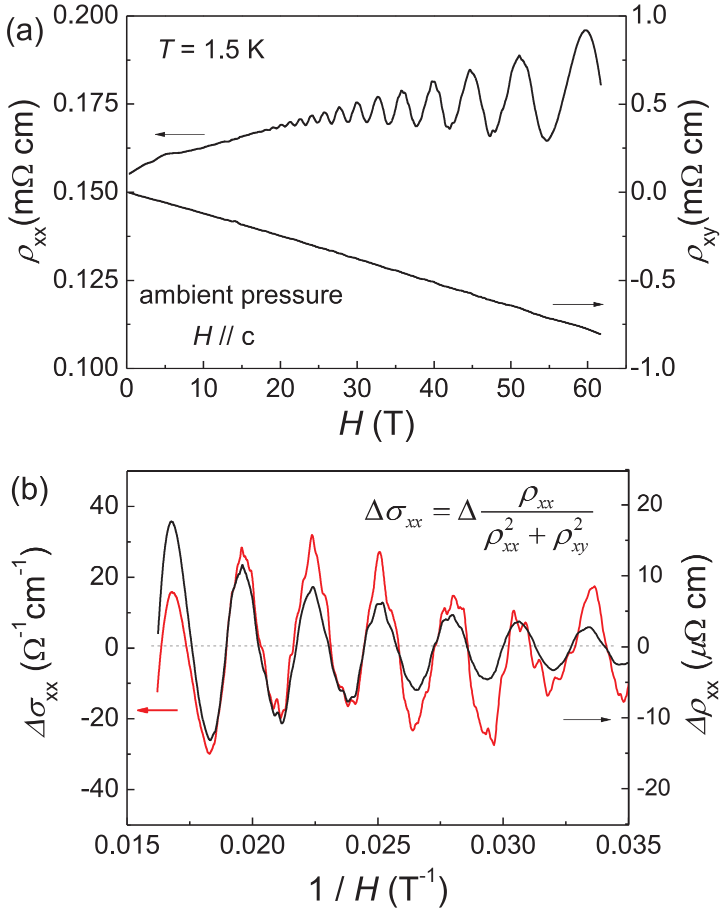

FIG. 4: (color online) (a) Longitudinal resistivity ρ_{xx} and the Hall resistivity ρ_{xy} as a function of applied magnetic field at ambient pressure. (b) Comparison between the quantum oscillations, $\Delta\sigma_{xx}$ and $\Delta\rho_{xx}$, where $\Delta\sigma_{xx}$ is given by $\Delta\sigma_{xx} = \Delta\rho_{xx}/(\rho_{xx}^2+\rho_{xy}^2)$. $\Delta\sigma_{xx}$ and $\Delta\rho_{xx}$ are in-phase indicating that phase of quantum oscillations are independent of carrier mixing between ρ_{xx} and ρ_{xy}.
